# Global Longitudinal Strain as a Sensitive Marker of Left Ventricular Dysfunction in Pediatric Dilated Cardiomyopathy: A Case–Control Study

**DOI:** 10.3390/jcdd12090351

**Published:** 2025-09-12

**Authors:** Iolanda Muntean, Beatrix-Jullia Hack, Asmaa Carla Hagau

**Affiliations:** 1 Department of Paediatrics III, George Emil Palade University of Medicine, Pharmacy, Science, and Technology of Târgu Mureș, 540136 Targu Mures, Romania; iolanda.muntean@umfst.ro; 2 Clinic of Pediatric Cardiology, Emergency Institute for Cardiovascular Diseases and Transplantation of Targu Mureș, 540139 Targu Mures, Romania; beatrix_hack@yahoo.com; 3 Pediatric Clinical Hospital, 550106 Sibiu, Romania; 4 Doctoral School of Medicine and Pharmacy, George Emil Palade University of Medicine, Pharmacy, Science, and Technology of Targu Mures, 540136 Targu Mures, Romania

**Keywords:** pediatric dilated cardiomyopathy, speckle-tracking, global longitudinal strain, left ventricular dysfunction, myocardial deformation

## Abstract

Pediatric dilated cardiomyopathy (DCM) is a rare but important cause of heart failure (HF) and a major indication for cardiac transplantation. Early detection of subclinical myocardial dysfunction is essential for risk stratification and management. This study aimed to evaluate left ventricular (LV) systolic function in children with DCM using conventional echocardiographic parameters and speckle-tracking echocardiography (STE) and to explore the relationship between deformation indices, clinical severity and biomarkers. **Methods:** We conducted a case–control study including 29 children diagnosed with DCM and 27 healthy controls matched by age and sex. All participants underwent clinical evaluation, NT-proBNP measurement, and transthoracic echocardiography. LV systolic function was assessed using conventional echocardiographic parameters, while STE was used to measure LV global longitudinal strain (GLS) and strain rate (SR) from all apical views. **Results:** GLS and SR were significantly reduced in the DCM group across all apical views (Global GLS: −11.13 ± 6.79% vs. −19.98 ± 3.25%, Global SR: −0.74 ± 0.39 s^−1^ vs. −1.12 ± 0.16 s^−1^; *p* < 0.01). GLS strongly correlated with functional indices (LV ejection fraction, shortening fraction, S′ lateral wave), LV end-diastolic diameter Z-score and NT-proBNP (*p* < 0.05), but not with MAPSE. In the primary model, GLS was associated with NYHA/Ross III–IV (OR 1.54 per 1% increase; 95% CI 1.14–2.07; *p* = 0.005); adding systolic blood pressure (*p* = 0.798) or heart rate (*p* = 0.973) did not materially change the GLS estimate (Δ ≤ 2%). In separate collinearity-avoiding models, LVEF (OR 1.12 per 1% decrease; 95% CI 1.03–1.22; *p* = 0.009), LVSF (OR 1.19 per 1% decrease; 95% CI 1.04–1.36; *p* = 0.011), and NT-proBNP (≈OR 1.11 per 100 units; *p* = 0.013) were also associated with advanced class. ROC analysis showed excellent discrimination for NT-proBNP (AUC 0.948) and GLS (AUC 0.906), and good–excellent performance for LVEF (AUC 0.869) and LVSF (AUC 0.875). **Conclusions:** Speckle-tracking derived parameters such as GLS and SR are sensitive and clinically relevant markers of LV dysfunction in pediatric DCM. Global longitudinal strain demonstrated a strong association with both clinical and biochemical markers of disease severity, after accounting for heart rate and blood pressure, supporting its integration into routine evaluation and risk stratification in pediatric DCM.

## 1. Introduction

Pediatric cardiomyopathies are uncommon disorders in children. Among the various types, dilated cardiomyopathy (DCM) makes up 50–60% of cases, with an incidence of 0.003–0.006% in children and adolescents and 0.05% in infants [1]. Compared to adults, DCM is much less common in children, possibly because they have less exposure to environmental risk factors and because genetically driven heart changes often manifest later in life [2]. However, despite its lower frequency, DCM leads to higher morbidity in children, as affected children usually develop progressive heart failure (HF), with decreased functional capacity and a high risk of adverse outcomes such as heart transplantation [3].

Dilated cardiomyopathy in the paediatric population results from either genetic mutations or cardiac injury, triggering a cascade of neurohormonal and structural changes that eventually impair left ventricular (LV) systolic function: the primary mechanism in paediatric HF. Compensatory responses, such as activation of the Frank-Starling mechanism and elevated heart rate (HR), initially preserve cardiac output (CO); however, ongoing volume and pressure overload lead to progressive cardiomyocyte necrosis accompanied by inflammatory infiltration. These alterations promote pathological cardiac remodelling characterised by myocyte disarray and ultimately myocardial fibrosis, a hallmark of advanced DCM [4]. Cardiac remodelling involves not just structural distortion but also changes in ventricular geometry, increased wall stress and fibrotic replacement of viable myocardium [5]. Interestingly, paediatric hearts, especially those of infants, possess a greater regenerative capacity and lower fibrosis compared to adult hearts; thus, early myocardial contractile abnormalities can occur without significant changes in conventional echocardiographic parameters [6]. Consequently, such subclinical alterations can expose the limitations of traditional echocardiographic measures of systolic function. For instance, LV ejection fraction (LVEF) has limitations as a sole marker for LV systolic performance due to load-dependent factors (preload, afterload, and HR), which restrict its utility in combined systolic and diastolic dysfunction [7].

The diagnosis of paediatric DCM relies on clinical signs of HF and echocardiographic criteria. Clinically, presentation varies widely with age: some patients remain asymptomatic for extended periods, while others (particularly infants) show signs of HF early in disease progression [8]. Heart failure severity is assessed using the Ross classification for children under 5 years and the New York Heart Association (NYHA) classification for older children [3]. On the imaging front, echocardiographic diagnosis of DCM is based on evidence of LV dilatation with associated systolic dysfunction, defined by a reduced LVEF ≤ 50% or a Z-score above 2 standard deviations (SD), an LV fractional shortening (LVFS) ≤ 25%, and/or an increased LV end-diastolic diameter (LVEDD) or volume (LVEDV) with a Z-score above 2 SD [8].

Among all imaging modalities, transthoracic echocardiography remains the first-line diagnostic tool in pediatric DCM due to its wide availability, accessibility, non-invasive nature and real-time capability [8]. However, despite its widespread use, conventional echocardiography parameters have important limitations for detecting early contractile dysfunction and in cases with severe dyssynchrony [9]. Given these limitations, recent studies have emphasised the role of two-dimensional speckle-tracking echocardiography (2D-STE) parameters such as myocardial strain, which can detect subclinical dysfunction and provide deeper insights into myocardial mechanics. In the adult population, 2D-STE demonstrated good results in the assessment of both global and regional cardiac function across all chambers, as well as in the early detection of subclinical ventricular dysfunction [10]. Furthermore, the use of STE in adults is recommended by the European Association of Cardiovascular Imaging (EACVI) and supported by expert consensus documents from the EACVI and American Society of Echocardiography (ASE) for the quantitative evaluation of LV mechanics [11,12]. However, in the pediatric population, the studies are still scarce.

Therefore, this study aimed to assess global and regional myocardial deformation in pediatric patients with DCM using 2D-STE. Additionally, the study sought to investigate the relationship between STE parameters and conventional echocardiographic parameters of LV function, to evaluate the added diagnostic and pathophysiological value of the strain imaging in the assessment of pediatric DCM.

## 2. Materials and Methods

This observational case–control study was conducted at the Pediatric Cardiology Clinic, part of the Institute of Cardiovascular Diseases and Transplantation in Târgu Mureș, Romania, between January 2022 and December 2023. Before enrollment, the study protocol was thoroughly explained to the parents or legal guardians of all participants. Written informed consent was obtained for both participation in the study and the anonymous publication of the collected data. The study was carried out following the principles of the Declaration of Helsinki and was approved by the institutional Ethics Committee (approval number 7697/2020).

### 2.1. Patient Sample Used

This study included 29 pediatric patients aged between 1 month and 18 years diagnosed with primary DCM and followed at the Pediatric Cardiology Clinic of the aforementioned Institute (DCM Group). The diagnosis of DCM was established based on an increased LVEDD with a Z-score ≥ 2 SD. Inclusion criteria were an age below 18 years and a diagnosis of primary DCM confirmed echocardiographically, after exclusion of secondary causes. Exclusion criteria were age above 18 years and secondary forms of DCM due to hypertension, congenital heart diseases, coronary artery anomalies, valvular disease, neuromuscular, metabolic, infectious or systemic disorders.

Given the case–control design of the study, data from the DCM group were compared with those of a control group comprising 27 healthy subjects without a history of cardiovascular disease, matched for residential background, ethnicity, age, sex and anthropometric characteristics (Control Group).

### 2.2. Working Method

All enrolled patients underwent clinical evaluation, measurement of N-terminal pro-B-type natriuretic peptide (NT-proBNP), and comprehensive transthoracic evaluation.

#### 2.2.1. Clinical Assessment

Before laboratory testing and echocardiographic examination, each patient underwent a thorough physical examination conducted by a pediatric cardiologist. Clinical data recorded included vital signs and anthropometric measurements. The severity of HF was assessed using age-appropriate clinical scoring systems: the Ross classification for children under 5 years of age and the New York Heart Association (NYHA) functional classification for older children and adolescents, consistent with pediatric HF practice [13,14]. For analytic purposes, we harmonised these scales into a binary variable: mild (Class I–II) versus severe (Class III–IV).

#### 2.2.2. Blood Sampling

Venous blood samples were collected from both patients and controls on the same day as the echocardiographic examination was performed. Serum NT-proBNP was determined using a chemiluminescent immunoassay, performed on serum samples and analysed using a fully automated analyser (Roche Cobas, Mannheim, Germany), following standard laboratory protocols. After obtaining NT-proBNP values for each patient and control, the log-transformed Z-score (logZ NT-proBNP) was calculated using the online calculator provided by the German Heart Centre in Munich, which adjusts for age- and sex-specific reference values in the pediatric population [15,16].

#### 2.2.3. Echocardiography

Transthoracic echocardiographic examinations were performed using a Philips EPIQ 7 ultrasound system equipped with 5–12 MHz transducers. All studies were acquired and analysed according to the recommendations of the American Society of Echocardiography (ASE) regarding the assessment and quantification of pediatric echocardiographic evaluations, as well as the standardised image acquisition and measurement protocol for pediatric patients [17]. No sedation was required for any of the examinations.

Left ventricular systolic function was assessed using the following parameters: mitral annular plane systolic excursion (MAPSE), LVEF, LVFS, interventricular septum (IVS) thickness in systole and diastole, LV end-systolic diameter (LVESD) and LVEDD, stroke volume (LVSV), CO and S’ lateral wave velocity (obtained via pulsed-wave Tissue Doppler imaging at the lateral mitral annulus). The z-scores for echocardiographic measurements were calculated using Cantinotti’s pediatric reference nomograms [18].

#### 2.2.4. Speckle-Tracking Analysis

Left ventricular deformation parameters were evaluated using 2D-STE. Offline analysis was performed using QLAB Cardiac Analysis software version 10 (Philips Healthcare, Andover, MA, USA), using the TomTec AutoStrain module, which enables semi-automated endocardial border detection and ST analysis across standard apical views.

Two-dimensional images were obtained from the apical four-chamber (A4C), two-chamber (A2C), and three-chamber (A3C) views, using a frame rate of 70–80 Hz. Although frame rates above 90 may be advantageous in smaller patients with higher HR, previous studies have demonstrated that the reproducibility of LV global longitudinal strain (LV GLS) is optimal when acquisition rates are between 60 and 90 frames per second; therefore, our chosen frame rate is considered suitable for STE in the pediatric population [19,20]. Sector width and image depth were optimised to maximise temporal resolution while ensuring complete visualisation of the LV myocardium. All images were stored in Digital Imaging and Communications in Medicine (DICOM) format. For each recorded acquisition, three consecutive cardiac cycles were selected for the analysis, with the software automatically identifying the cardiac cycle.

**Figure 1:**
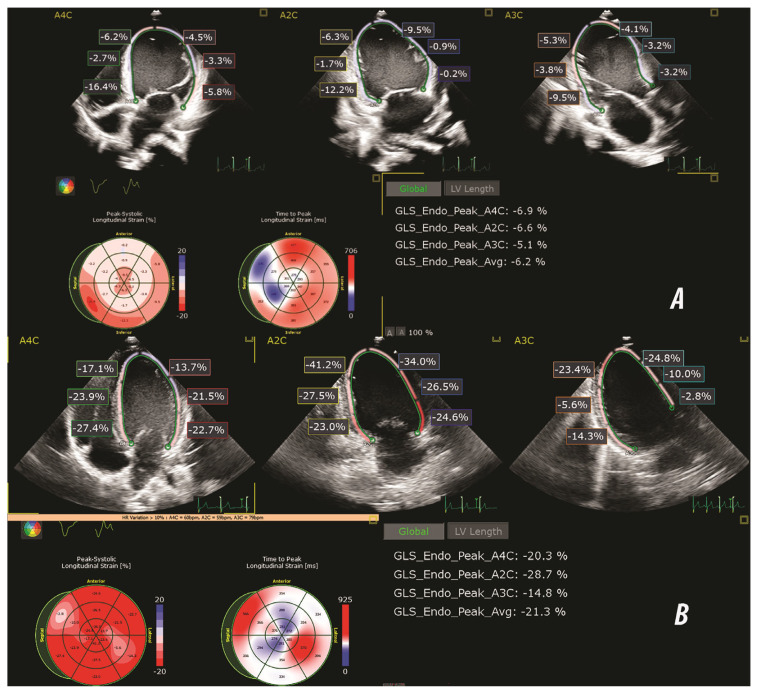
Representative speckle-tracking echocardiography images illustrating global longitudinal strain (GLS) analysis in a patient with DCM (**A**) and a healthy control subject (**B**). Longitudinal strain was assessed using apical 4-chamber (A4C), two-chamber (A2C) and three-chamber (A3C) views. The bull's eye plots display segmental peak systolic strain (%) and time to peak strain (msec). The strain values shown in each panel reflect endocardial peak systolic strain (GLS Endo Peak) for each apical view, with the averaged global value.

After marking the three anatomical points on the endocardial border (basal septal, basal lateral, and apical points), the software automatically generated the endocardial border. Subsequently, the borders were manually adjusted to ensure accurate tracking during the cardiac cycle and to eliminate tracking artefacts. Global longitudinal strain of the LV and strain rate (SR) were calculated as the average peak systolic strain of all analyzable segments, from the three mentioned apical views. The global values were derived as the mean of segmental peak systolic strain, following the 18-segment LV module implemented in QLAB 10 with the TomTec Autostrain module, which included additional apical segments to enhance regional deformation analysis (Figure 1). TomTec algorithms have demonstrated feasibility and reproducibility in pediatric cohorts [21].

For regional strain assessment, the 18-LV segments obtained from the 2D-STE analysis were grouped into three anatomical levels- basal, mid-ventricular and apical. The basal level comprised 6 segments adjacent to the atrio-ventricular plane, the mid-ventricular level included 6 segments at the papillary muscle level, and the apical level encompassed the remaining 5 apical wall segments plus the apical cap. Peak systolic longitudinal strain values for all segments were averaged to obtain the corresponding regional strain values > basal longitudinal strain (BLS), mid-ventricular longitudinal strain (MLS) and apical longitudinal strain (ALS).

### 2.3. Statistical Tests Used

Statistical analysis was performed using GraphPad Prism 10.0 (GraphPad Software, San Diego, CA, USA), IBM SPSS Statistics version 13.0 (IBM Corp., Armonk, NY, USA), and Microsoft Excel. A two-tailed *p*-value < 0.05 was considered statistically significant.

The normality of data distribution was assessed for all continuous variables using appropriate tests such as the Shapiro–Wilk test. Continuous variables were expressed as mean ± standard deviation (SD) for parametric distributions or median ± interquartile range (IQR) for non-parametric distributions. Categorical variables were summarised as absolute frequencies and percentages.

Group comparisons between the DCM and control group, as well as between a subgroup of the DCM cohort, were performed using unpaired Student’s *t*-tests for parametric data and Mann–Whitney U tests for non-parametric data.

Correlation analyses were performed using Pearson’s correlation coefficient for parametric variables and Spearman’s rank correlation coefficient for non-parametric variables.

The primary outcome was advanced functional class, defined as NYHA/Ross III–IV versus I–II. Global longitudinal strain (GLS; more positive/less negative values indicate worse systolic function) was modelled as a continuous predictor in a parsimonious logistic regression. Given the known collinearity between deformation indices and conventional systolic parameters (LVEF and LVSF) and NT-proBNP (raw values), they were evaluated in separate alternative models. To assess robustness to potential hemodynamic confounding, hierarchical sensitivity models added, separately, HR and systolic BP to the GLS model; a change < 10% in the GLS coefficient relative to the primary model was interpreted as negligible confounding. Model calibration was assessed using the Hosmer–Lemeshow test; classification accuracy at a 0.5 threshold is reported for transparency. Because pediatric strain varies with age and BMI, we ran blockwise sensitivity models adding age and BMI to the GLS-only logistic regression; adjusted ORs (95% CI), Hosmer–Lemeshow *p*, accuracy, and per cent change in the GLS coefficient versus the primary model were reported.

Receiver operating characteristic (ROC) analyses were performed for GLS, LVEF, LVSF, and NT-proBNP (direction oriented so that larger values indicate the positive state; EF/SF analysed accordingly), and for predicted probabilities from the GLS, LVEF, LVSF and NT-proBNP-only models. Areas under the curve (AUCs) with 95% confidence intervals (CIs) are presented.

## 3. Results

### 3.1. Baseline Characteristics of the Study Population

A total of 29 patients diagnosed with DCM were included in the case group. The baseline characteristics of the study population are presented in Table 1. The DCM group comprised 21 males and 8 females, with ages ranging from infancy to adolescence.

The median age in the DCM group was 10.5 years (IQR, 3.5–16 years), while in the Control group, it was 13.5 years (IQR, 10.9–17.6). The sex distribution in both groups showed a higher proportion of males, with a male-to-female ratio of approximately 2:1. There were no statistically significant differences between groups regarding residential background, age, or anthropometric measures (weight, height, and BMI—body mass index).

Compared to controls, children with DCM had significantly lower absolute systolic blood pressure (BP) values (102.9 ± 11.7 vs. 107.9 ± 9.5 mmHg, *p* < 0.01) and diastolic BP values (60.2 ± 11.0 vs. 70.4 ± 9.6 mmHg, *p* < 0.01). However, when expressed as age-, sex-, and height-adjusted percentiles, systolic (48.4 ± 29.8 vs. 47.1 ± 24.0, *p* = 0.87) and diastolic BP (56.0 ± 26.1 vs. 66.2 ± 24.6, *p* = 0.14), there were no significant differences between groups. The absence of differences in systolic and diastolic BP percentiles suggests comparable BP relative to age/sex/height norms. The DCM group had a higher HR compared to the control group (87.5 ± 17.0 vs. 79.6 ± 13.6 bpm), but this was not statistically significant (*p* = 0.063).

According to the NYHA/Ross classification, in the DCM group, 17,2% (no. 5) were classified as NYHA/Ross Class I, 27.5% (no. 8) as NYHA/Ross Class II, 34.4% (no. 10) as NYHA/Ross Class III and 20.6% (no. 6) as NYHA/Ross Class IV. As expected, serum NT-proBNP levels and zLog NT-proBNP were significantly higher in the DCM group compared to the Control group, indicating a higher degree of myocardial stress and HF severity in DCM patients.

### 3.2. Comparison of Conventional Echocardiographic Parameters Between the DCM and Control Groups

When we compared conventional echocardiographic parameters related to LV structure and function, significant differences were observed between the DCM and Control groups (Table 2).

The DCM group showed markedly reduced systolic function, with significantly lower MAPSE (1.02 ± 0.32 cm vs. 1.71 ± 0.27 cm, *p* < 0.01), LVEF (29.42 ± 14.35% vs. 72.17 ± 4.78%, *p* < 0.01), and LV SF (22.79 ± 9.89% vs. 41.26 ± 4.19%, *p* < 0.01). Additionally, LV SV (31.27 ± 19.41 mL vs. 52.07 ± 15.7 mL) and LV CO (2.47 ± 1.12 L/min vs. 4.04 ± 1.17 L/min) were significantly lower in the DCM group (*p* < 0.01 for both).

Regarding LV dimensions, both LVEDD and LVESD were significantly increased in DCM patients (LVEDD: 6.77 ± 6.41 cm vs. 4.35 ± 0.6 cm; LVEDD Z-score: 4.47 ± 2.42 vs. −3.31 ± 1.61; LVESD: 4.42 ± 1.59 cm vs. 2.55 ± 0.38 cm; *p* < 0.01). Furthermore, LV IVS and LV PW thicknesses during both systole and diastole showed significant differences between the groups (*p* < 0.01).

### 3.3. Global Myocardial Deformation- Global Longitudinal Strain and Strain Rate

**Figure 2:**
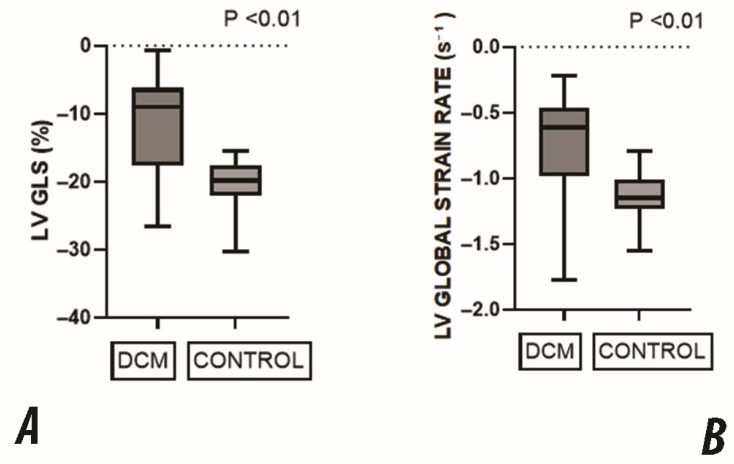
Comparison of global LV deformation parameters between DCM and the control group. (**A**) Global Longitudinal Strain (GLS) was significantly reduced in the DCM group, reflecting impaired systolic function. (**B**) Global Strain Rate (SR) was also significantly lower in DCM patients, indicating decreased myocardial contractility. All values are expressed as medians with interquartile ranges. All comparisons showed statistically significant differences (*p* < 0.01).

Global longitudinal strain was significantly reduced in the DCM group compared to the control group in all apical views (Table 3, Figure 2). Similarly, the global SR was significantly lower in the DCM group across all views.

These results confirm a marked impairment in both the magnitude and rate of myocardial deformation in children with DCM, consistent across all standard apical views.

**Figure 3:**
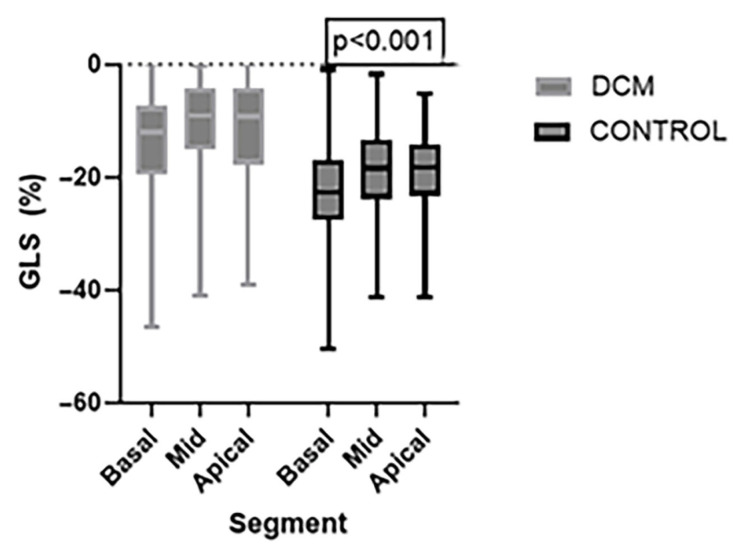
Comparison of regional Global Longitudinal Strain (GLS) values between the DCM and Control group at the basal, mid and apical levels of the LV. Strain values were significantly reduced in the DCM group across all myocardial regions. Data are presented in the box plots as median and interquartile range.

Regional analysis of longitudinal strain revealed significantly reduced strain values in all myocardial levels in the DCM group compared to the Control group. Specifically, the basal segment was markedly decreased (−13.4 ± 8.54 in the DCM group vs. −22.41 ± 9.22 in the Control Group), as was mid-ventricular strain (−10.51 ± 7.75 in the DCM group vs. −18.6 ± 7.59 in the Control Group) and apical strain (−11.76 ± 9.73 in the DCM group vs. −19.01 ± 6.34 in the Control Group). These findings indicate a global impairment in the longitudinal myocardial deformation in all segments in DCM patients, suggesting diffuse systolic dysfunction. However, the most severe impairment appears in the mid region, disturbing the normal base-to-apex gradient (Figure 3).

### 3.4. Correlation Between Global Longitudinal Strain and Functional and Biomarker Parameters

**Figure 4:**
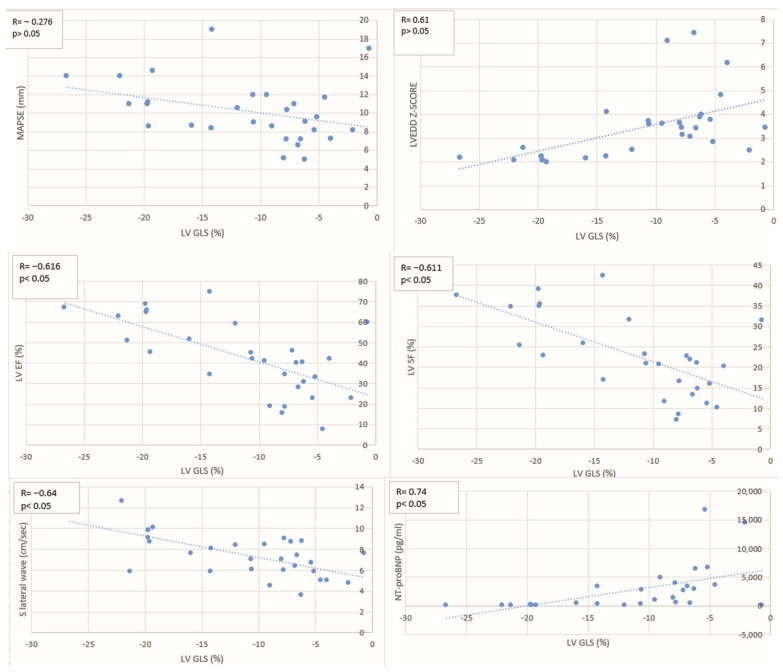
Scatter plots illustrate the relationship between GLS and MAPSE, LVEDD Z-score, LVEF, LVSF, lateral S′ wave, and NT-proBNP levels. Each plot includes the correlation coefficient (R) and *p*-value. Abbreviations: GLS—global longitudinal strain; MAPSE—mitral annular plane systolic excursion; LVEDD—left ventricular end-diastolic diameter; LVEF—left ventricular ejection fraction; LVSF—left ventricular shortening fraction; S′—lateral mitral annular systolic velocity; NT-proBNP—N-terminal pro–B-type natriuretic peptide.

A significant inverse correlation was observed between LV GLS and both LVEF (R = −0.616, *p* < 0.05) and LVSF (R = −0.611, *p* < 0.05), suggesting that impaired longitudinal strain is associated with a decline in the systolic function (Table 4, Figure 4). Similarly, a significant inverse correlation was found with the lateral S′ wave on tissue Doppler imaging (R = −0.64, *p* < 0.05), further supporting the link between reduced myocardial deformation and impaired longitudinal mechanics. A moderate positive correlation was also observed between LV GLS and LVEDD Z-score (R = 0.61, *p* < 0.05), suggesting that increased ventricular dilatation is associated with worsening strain. In addition, LV GLS showed a strong positive correlation with NT-proBNP levels (R = 0.74, *p* < 0.05), reflecting the relationship between myocardial dysfunction and elevated cardiac biomarkers. However, when we analysed the correlation between LV GLS and MAPSE, although a negative correlation was also observed, it did not reach statistical significance (R = −0.28, *p* > 0.05), likely due to the age-dependence of MAPSE values and their variability in the pediatric population.

### 3.5. Subgroup Analysis

To evaluate the association between disease severity and cardiac function, the DCM cohort was stratified according to NYHA/Ross functional class: patients with mild symptoms (NYHA/Ross class I–II; no. 13) were compared to those with moderate or severe symptoms (NYHA/Ross class III–IV; no. 16). Significant differences were observed between the two subgroups (Table 5). NT-proBNP levels were markedly elevated in patients with moderate/severe symptoms (Median 3500 pg/mL, IQR 1706–56880) compared to those with mild symptoms (Median 119.5 pg/mL, IQR 77.5–371.3; *p* < 0.001). Similarly, conventional systolic function parameters such as LVEF (21.68 ± 11.86% vs. 38.28 ± 11.48%, *p* < 0.001), LVSF (17.65 ± 8.74% vs. 28.22 ± 7.87%, *p* < 0.001) and MAPSE (0.88 ± 0.2 cm vs. 1.17 ± 0.38 cm, *p* < 0.05) were significantly reduced in the NYHA/Ross III–IV group. Global longitudinal strain was also more severely impaired in patients with more severe symptoms (−7.41 ± 2.98% vs. −16.59 ± 5.78%, *p* < 0.001), indicating reduced myocardial deformation capacity. Also, LVEDD Z-scores were higher in the more symptomatic group (4.09 ± 1.53 vs. 2.71 ± 0.70, *p* < 0.001), indicating greater chamber dilatation in more advanced disease.

### 3.6. Primary Model

Global longitudinal strain was significantly associated with advanced functional class (OR 1.54 per 1% increase—i.e., less negative GLS; 95% CI 1.14–2.07; *p* = 0.005). Calibration was adequate (Hosmer–Lemeshow *p* = 0.704), with an overall classification accuracy of 89.3% (sensitivity 93.8%, specificity 83.3%). Adding systolic BP or HR yielded a nearly identical GLS estimate (OR 1.55 and 1.53, respectively; change in the GLS coefficient was ≤2%, Hosmer–Lemeshow *p* = 0.560–0.698, accuracy 88.9%). Also, we ran another sensitivity model that included GLS, age, and BMI. GLS remained significantly associated with NYHA/Ross III–IV (OR 1.51; 95% CI 1.07–2.14; *p* = 0.018), with excellent calibration (Hosmer-Lemeshow *p* = 0.841) and accuracy 85.7% (Table 6).

Evaluated separately to avoid collinearity, LVEF (OR 1.12 per 1% ↓; 95% CI 1.03–1.22; *p* = 0.009), LVSF (OR 1.19 per 1% ↓; 95% CI 1.04–1.36; *p* = 0.011), and NT-proBNP (OR ≈ 1.11 per 100 units ↑; 95% CI ≈ 1.00–1.22; *p* = 0.013) were each associated with NYHA/Ross III–IV (Table 7).

**Figure 5:**
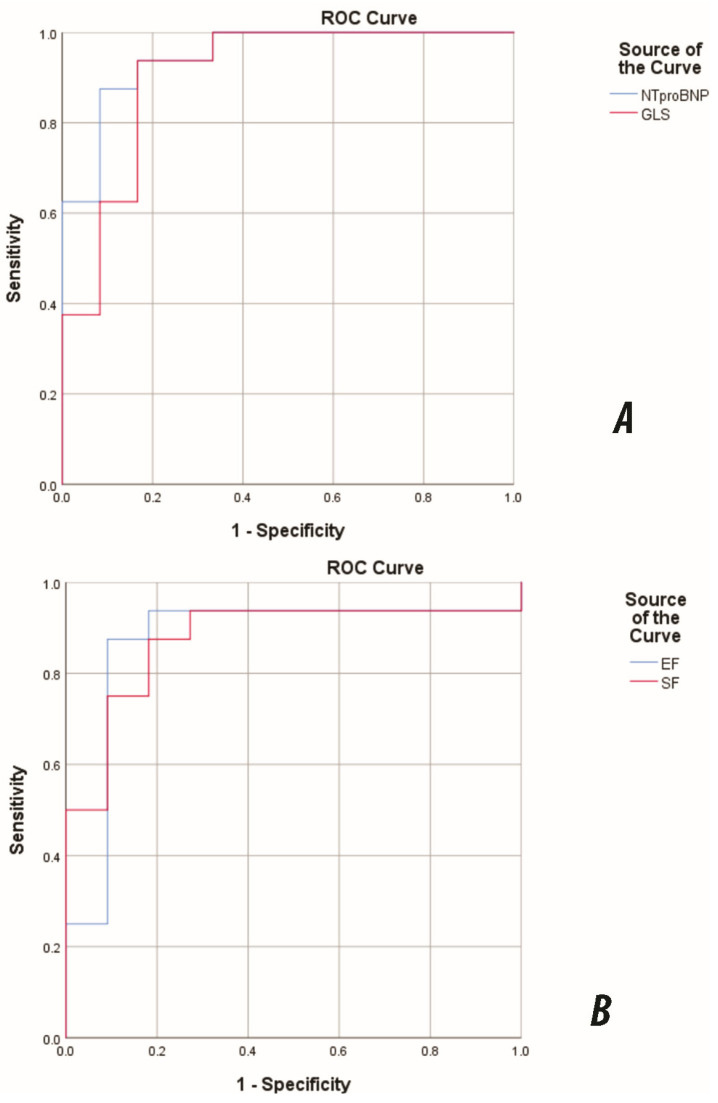
ROC curves for discrimination of advanced functional class (NYHA/Ross III–IV) in pediatric DCM. (**A**) NT-proBNP and GLS; (**B**) EF and SF. NT-proBNP AUC = 0.948 (95% CI 0.871–1.000); GLS AUC = 0.906 (95% CI 0.786–1.000); EF AUC = 0.869; SF AUC = 0.875. The direction was set so that larger values indicated the positive state; EF/SF were analysed accordingly. Abbreviations: DCM, dilated cardiomyopathy. AUC, area under the curve; GLS, global longitudinal strain; EF, ejection fraction; SF, shortening fraction.

On ROC analysis, NT-proBNP and GLS showed excellent discrimination for NYHA/Ross III–IV (AUC 0.948, 95% CI 0.871–1.000; and 0.906, 0.786–1.000; both *p* < 0.001), while EF and SF demonstrated good–excellent discrimination (AUC 0.869, 0.706–1.000; and 0.875, 0.732–1.000; both *p* = 0.001) (Figure 5).

## 4. Discussion

This study provides a detailed evaluation of LV systolic performance in pediatric patients with DCM, using both conventional echocardiographic parameters and 2D-STE strain-derived measures.

In our paediatric DCM cohort, most patients were male (72%), with a mean age of 10.53 ± 6.7 years. Previous studies have shown that male sex is an independent risk factor for adverse events such as sudden cardiac death and progressive HF, while female patients tend to have better outcomes, including lower mortality rates and a more favourable response to HF therapy [8]. Additionally, the patient’s age at diagnosis strongly indicates prognosis: children diagnosed after the age of 6 years have a 3 to 4-fold increased risk of death or cardiac transplantation compared to those diagnosed before 2 years of age [22].

As expected, the patients with DCM exhibited a significant decrease in LVEF, LVFS, and MAPSE. Also, they demonstrated increased LV diameters and Z-scores compared to the controls. These findings confirm the presence of global systolic dysfunction and LV remodelling. To perform a more comprehensive evaluation of myocardial function, STE was utilised. The myocardium is composed of a complex arrangement of three primary fibre types (longitudinal, radial, and circumferential), which facilitate ventricular contraction. Among these, the longitudinal fibres are particularly vulnerable to early myocardial injury [10]. Apical long-axis echocardiographic views, which are used for longitudinal strain assessment, provide superior spatial resolution and include a larger part of the myocardial wall compared to short-axis views [23,24]. Therefore, this study focused on evaluating global and regional GLS, as considered the most sensitive and well-validated measure of myocardial deformation [25].

The DCM group showed a significant reduction in GLS across all apical views, with a mean global average of −11.13 ± 6.79%. These observations also align with prior pediatric studies, which documented early systolic dysfunction in children with DCM, even when ventricular geometry was preserved or LVEF was at the borderline level. In contrast, the healthy control population presented with GLS values of −19.98 ± 3.25, a finding consistent with previously published studies [26,27].

In the control group of our cohort, the expected base-to-apex gradient was partially observed, with the basal segments exhibiting the highest magnitude of deformation, followed by the apical and mid-ventricular segments. This pattern is inconsistent with some previous reports. For instance, Chatterjee et al. found no significant increase in GLS from basal to apical segments across pediatric age groups between 2 and 15 years old [28]. In contrast, Marcus et al. described a base-to-apex increase in LV strain in healthy pediatric patients [29]. These discrepancies may suggest that methodological differences or sample characteristics may influence the magnitude of the base-to-apex gradient in healthy pediatric patients. In contrast, in our cohort of patients with DCM, regional longitudinal strain analyses demonstrated that the most significant impairment occurred in the mid-ventricular and apical regions of the heart. Therefore, the DCM group displayed an irregular strain gradient, with a similarly substantial degree of impairment observed across all myocardial levels. These findings are consistent with diffuse systolic dysfunction. The segmental pattern of myocardial dysfunction is consistent with the results of the few studies that provided segmental analysis. For instance, Forsha et al. noted that abnormal segmental strain in pediatric DCM patients was associated with poorer clinical outcomes, including an increased risk of heart transplantation and mortality [30]. Furthermore, Van der Meulen et al. highlighted the prognostic value of tracking segmental strain measurements over time to monitor disease progression in pediatric DCM [31]. Additionally, studies using cardiac magnetic resonance imaging in adult DCM patients, such as that published by Yu et al., have also reported a significant decrease in the strain values throughout the myocardium, supporting a diffuse pattern of myocardial involvement [32]. These findings highlight the potential of regional strain analysis as a sensitive tool for detecting subtle myocardial dysfunction and as a non-invasive marker of the severity of myocardial remodelling. Our findings emphasise the importance of incorporating segmental strain evaluation into the standard echocardiographic assessment for pediatric DCM, as conventional echocardiographic parameters may fail to identify clinically relevant regional dysfunction.

An additional significant finding was that the DCM cohort had significantly lower LVSR across all apical views in comparison to the control group. Strain rate is a parameter that reflects the velocity of myocardial deformation over time. Because it is less susceptible to the influence of preload or afterload, it is recognised as a sensitive parameter for detecting subtle contractile impairments [33,34]. In the paediatric population, because the haemodynamic variables often fluctuate, SR may be a more reliable indicator of myocardial contractility. Moreover, SR maintains greater stability across different HR, which is particularly beneficial in children, as their HR vary with age. A study that investigated the effect of HR on strain and SR in paediatric patients found that, while both parameters change with age, SR demonstrated a significantly lower dependency on heart rate, making it a more reproducible parameter in tachycardic states [35]. The observed reduction in SR within our DCM group suggests an increased contraction time, indicating a delayed mechanical response. Researchers have shown that SR in adult patients correlates more strongly with invasive measures of contractility and myocardial workload than does GLS [8].

Mitral annular plane systolic excursion is recognised as a simple and valuable tool for evaluating LV systolic function in the adult population [36,37]. In the pediatric population, however, available data remain limited. In our cohort, MAPSE values were significantly reduced in the DCM group compared to the control, supporting its potential utility as a sensitive marker for detecting systolic dysfunction in pediatric patients with DCM. These findings align with the existing literature, which supports the application of MAPSE in pediatric cardiac assessment [38,39]. While some studies have reported a correlation between MAPSE and GLS in adults with mild to moderate DCM, we did not observe a statistically significant correlation between these two parameters in our DCM cohort [40]. This contrasts with previous pediatric studies. For example, in a study published by Agha et al., they reported a significant correlation between MAPSE and GLS while assessing early ventricular dysfunction after chemotherapy in a pediatric cohort [41]. Similarly, Terada et al. demonstrated an association between MAPSE and GLS in a univariate analysis of healthy children [39]. The discrepancy in our findings may be attributed to a more severe degree of LV systolic dysfunction present in our patient population. Also, the lack of a significant correlation between MAPSE and GLS in our cohort study may be explained by several factors specific to paediatrics, such as the age-related and body-size variability of MAPSE [42]. These factors can limit its reproducibility in different pediatric populations in the context of advanced myocardial dysfunction. In contrast, GLS is less influenced by body size. Additionally, GLS provides a comprehensive measure of myocardial deformation across the entire LV, whereas MAPSE only assesses basal longitudinal motion. This difference may further explain the lack of correlation between the two parameters in our study.

A significant finding of the study reveals that GLS demonstrates a strong correlation with advanced functional class (NYHA/Ross III–IV). This association remained stable even after sensitivity adjustment for HR and systolic BP, with only a ≤2% variation in the GLS coefficient, while maintaining both calibration and accuracy. Children with DCM exhibited notably lower absolute systolic and diastolic BP values compared to the control group. However, when adjusted for percentiles, the BP values did not show significant differences between the two groups. Additionally, HR in the DCM group demonstrated only a non-significant trend towards higher values. These findings suggest that hemodynamic factors are unlikely to account for the relationship between impaired GLS and worse functional class. This supports the interpretation that impaired GLS primarily indicates intrinsic myocardial dysfunction. Our results are consistent with findings from adult studies, which have established GLS as a strong predictor of adverse outcomes in DCM, even when LVEF is preserved. These studies highlight the superior value of deformation imaging over traditional echocardiographic measures of LV systolic function [43,44]. Additionally, GLS measured via cardiac magnetic resonance in adults has been shown to have incremental predictive value for adverse events [45]. Limited pediatric literature also supports the use of GLS as a prognostic marker, with an independent association with the risk of heart transplant or death [8]. Because pediatric deformation varies with maturation and body mass, we also evaluated age and BMI as potential confounders. Previous meta-analyses and normative cohorts highlight significant age-related variations in LV GLS throughout childhood. Furthermore, additional inconsistencies arise from differences in segmentation, as well as variations in vendor and software [21,27,46,47]. As a result, the establishment of standardised z-scores remains incomplete. In our data, adding age or BMI did not materially alter the GLS–functional class association, indicating that the observed relationship is unlikely to be explained by age/body-mass differences alone.

Methodologically, we employed parsimonious GLS models with sensitivity adjustments and reported discrimination and calibration, while assessing LVEF, LVSF, and NT-proBNP in separate, collinearity-avoiding models, following approaches consistent with prior DCM research and recommended small-sample modelling practices [9,31,48]. In these alternative models, LVEF and LVSF showed the expected inverse association with severe functional class, emphasising that conventional parameters continue to track clinical impairment in paediatrics. Likewise, NT-proBNP showed a positive association with advanced class and excellent univariable discrimination. The ROC analysis was concordant: GLS achieved an excellent AUC (≈0.91), while LVEF and LVSF performed well (≈0. 87–0.88). These findings suggest that imaging and biomarkers offer complementary rather than interchangeable information: GLS assesses longitudinal fibre mechanics and diffuse remodelling, whereas NT-proBNP reflects wall stress and neurohormonal activation. High NT-proBNP AUCs in paediatric DCM have been reported in registry-based and institutional cohorts, whereas adult DCM cohorts consistently show incremental prognostic value when GLS is added to NYHA class and LVEF [49,50]. The present findings, therefore, align with both biomarker and deformation literature and support multimodal risk profiling.

Our study has several limitations. First, it is a single-centre, observational case–control study with a relatively small number of patients, which may constrain generalisability and reproducibility. However, pediatric DCM is a rare disease with low incidence, and recruiting larger cohorts remains difficult even in multicentre studies. Conducted at one of the largest paediatric tertiary centres in Romania and the only national centre performing paediatric heart transplants, our cohort included a diverse population with both early-stage and advanced HF cases. Additionally, although all enrolled patients had primary DCM, the relatively small sample size did not permit detailed subgroup analyses by aetiology, further limiting the generalisability of our findings. Second, while combining Ross and NYHA classifications into a dichotomous variable may introduce heterogeneity because the two scales are not entirely interchangeable, this approach reflects current practice in paediatric HF studies, where Ross is used for younger children and NYHA for older children to analyse functional status across a wider paediatric age range. Given our limited sample size, this pragmatic approach was suitable, although larger studies could enable stratified or ordinal analyses for a more detailed evaluation. Third, STE has known limitations, such as being time-consuming and requiring good image quality, which is often difficult to achieve in younger children. Moreover, vendor-specific differences in STE algorithms may cause variability in reported strain values. We also used an 18-segment LV model, as implemented by the QLAB 10 TomTec Autostrain module, rather than the standard 17-segment model recommended by ASE/EACVI. Although the additional apical segment enhances regional deformation assessment and may introduce minor discrepancies when comparing our results with other studies using the conventional 17-segment model, studies suggest that GLS values are generally consistent between the two approaches [11,12,21]. Another limitation of our study is the absence of a formal assessment of STE feasibility and reproducibility (intra-/interobserver variability). However, previous paediatric studies have demonstrated high feasibility and excellent reproducibility of STE-derived GLS in children [19,47,51]. Additionally, it is worth noting that the configuration of the anterior chest wall may artificially lower regional strain values. Sternal depression, in particular, has been linked to progressively lower myocardial strain, most notably at the basal segments, which is more likely due to compressive mechanics than intrinsic myocardial dysfunction [52]. We did not systematically quantify chest wall morphology; therefore, unmeasured confounding by chest wall cannot be ruled out.

However, these findings emphasise the diagnostic importance of STE in paediatric DCM. Our results suggest that GLS could be incorporated into standard echocardiographic assessment protocols for paediatric cardiomyopathy, providing additional value beyond conventional systolic indices. Due to the progressive nature of paediatric DCM, early detection of myocardial deformation using GLS may support earlier intervention, closer monitoring, or decisions regarding pharmacological or device therapy. Nevertheless, longitudinal studies are needed to determine whether GLS offers incremental prognostic value beyond LVEF/LVSF and NT-proBNP and to evaluate GLS-guided clinical pathways such as increased surveillance, therapy escalation, and timing of MCS/transplant referral. Including inter-/intra-observer reproducibility and load-challenge substudies would help clarify operation characteristics across paediatric haemodynamic states.

## 5. Conclusions

In conclusion, in this paediatric DCM cohort, LV GLS was strongly associated with advanced NYHA/Ross class and retained its association after adjustment for HR and systolic BP, indicating excellent discrimination of clinical severity. These findings support incorporating GLS alongside conventional echocardiographical systolic parameters and NT-proBNP into the routine evaluation of paediatric DCM, with validation in larger multicentre longitudinal cohorts.

## Figures and Tables

**Table 1:** Baseline characteristics of the DCM group versus the Control group.

	DCM Group (No. 29)	Control Group (No. 27)	*p*-Value
Residential Background			
Urban Area	41.37% (no. 12)	66.6% (no. 18)	0.07
Rural Area	58.6% (no. 17)	33.3% (no. 9)	0.08
Gender			
Male	72.41% (no. 21)	51.83% (no. 14)	0.16
Female	27.58% (no. 8)	48.14% (no. 13)	0.16
Age (yr)			
mean ± SD	10.53 ± 6.719	13.51 ± 4.22	0.17
Weight (kg)			
mean ± SD	44.29 ± 26.46	52.7 ± 16.36	0.33
Height (cm)			
mean ± SD	137.6 ± 47.68	158.8 ± 21.67	0.407
BMI (kg/m^2^)			
	18.2 ± 4.46	20.29 ± 2.88	0.06
NT-proBNP (pg/mL)	997 [119.5–56,880]	45 [35–185]	<0.001
zLog NT-proBNP	3.28 ± 2.12	0.36 ± 0.7	<0.001
Systolic BP (mmHg)	102.9 ± 11.72	107.9 ± 9.51	<0.001
Systolic BP percentile	48.44 ± 29.8	47.07 ± 24	0.87
Diastolic BP (mmHg)	60.19 ± 11	70.44 ± 9.58	<0.001
Diastolic BP percentile	56 ± 26.08	66.22 ± 24.57	0.14
HR (bpm)	87.5 ± 17.03	79.56 ± 13.62	0.063
NYHA/Ross Class			
I	17.2% (no. 5)		
II	27.5%(no. 8)		
III	34.4% (no. 10)		
IV	20.6% (no.6)		

No—number; yr—years; kg—kilograms; cm—centimetres; BMI—body mass index; NT-proBNP—N-terminal pro–B-type natriuretic peptide; BP—blood pressure; HR—heart rate; Bpm—beats per minute; NYHA—New York Heart Association. Data is presented as mean ± SD or median (IQR).

**Table 2:** Comparison of conventional echocardiographic parameters between the DCM and Control groups. Values are expressed as mean ± SD.

	DCM (Mean ± SD)	Control (Mean ± SD)	*p* Value
MAPSE (cm)	1.02 ± 0.32	1.71 ± 0.27	<0.01
IVS (cm)			
Systole	1.1 ± 0.46	1.35 ± 0.26	<0.01
Diastole	0.88 ± 0.31	0.93 ± 0.22	<0.01
LV PW (cm)			
Systole	1.08 ± 0.37	1.36 ± 0.29	<0.01
Diastole	1.1 ± 0.67	0.77 ± 0.25	<0.01
LVEDD (cm)	6.77 ± 6.41	4.35 ± 0.6	<0.01
LVEDD Z-SCORE	4.47 ± 2.42	−0.331 ± 1.61	<0.01
LVESD (cm)	4.42 ± 1.59	2.55 ± 0.38	<0.01
LV EF (%)	29.42 ± 14.35	72.17 ± 4.78	<0.01
LV SF (%)	22.79 ± 9.89	41.26 ± 4.19	<0.01
LV SV (mL)	31.27 ± 19.41	52.07 ± 15.7	<0.01
LV CO (L/min)	2.47 ± 1.12	4.04 ± 1.17	<0.01
S’ lateral	7.45 ± 2.14	11 ± 2.06	<0.01

Abbreviations: MAPSE—mitral annular plane systolic excursion; IVS—intraventricular septum; LV PW—left ventricle posterior wall; LVEDD—left ventricle end-diastolic diameter; LVEDD Z-score—left ventricular end-diastolic diameter Z-score; LVESD—left ventricle end-systolic diameter; LV EF—left ventricle ejection fraction; LV SF—left ventricle shortening fraction; LV SV—left ventricle stroke volume; LV CO—left ventricle cardiac output; cm—centimetres; mL—millilitres; L/min—litres/minute.

**Table 3:** Comparison of global longitudinal strain (GLS) and global strain rate (SR) between the DCM and Control groups. Values are expressed as mean ± SD. GLS and strain rate were measured in all standard apical views (A4C = apical four-chamber, A2C = apical two-chamber, A3C = apical three-chamber), and global values were calculated as the average of corresponding segmental values. Statistically significant reductions were observed in all strain and strain rate parameters in the DCM group compared to controls (*p* < 0.01 for all comparisons). Abbreviations: GLS—global longitudinal strain; SR—strain rate. s^−1^—per second; SD—standard deviation.

	DCM Group (Mean ± SD)	Control Group (Mean ± SD)	*p* Value
GLS A4C (%)	−12.14 ± 7.01	−20.73 ± 3.66	<0.01
GLS A2C (%)	−10.14 ± 8.65	−20.81 ± 4.58	<0.01
GLS A3C (%)	−11.10 ± 7.09	−18.41 ± 3.56	<0.01
Global GLS	−11.13 ± 6.79	−19.98 ± 3.25	<0.01
SR A4C (s^−1^)	−0.85 ± 0.53	−1.18 ± 0.25	<0.01
SR A3C (s^−1^)	−0.72 ± 0.46	−1.06 ± 0.24	<0.01
SR A2C (s^−1^)	−0.73 ± 0.49	−1.14 ± 0.25	<0.01
Global SR (s^−1^)	−0.74 ± 0.39	−1.12 ± 0.16	<0.01

**Table 4:** Correlation between global longitudinal strain (GLS), conventional echocardiographic parameters and laboratory parameters in the DCM group. Abbreviations: GLS—global longitudinal strain; MAPSE—mitral annular plane systolic excursion; LVEDD—left ventricular end-diastolic diameter; LVEF—left ventricular ejection fraction; LVSF—left ventricular shortening fraction; S′—lateral mitral annular systolic velocity; NT-proBNP—N-terminal pro–B-type natriuretic peptide.

Variable	R-Value	*p*-Value
MAPSE	−0.276	>0.05
LVEDD Z-score	0.61	<0.05
LV EF	−0.616	<0.05
LV SF	−0.611	<0.05
S lateral wave	−0.64	<0.05
NT-proBNP	0.74	<0.05

**Table 5:** Comparison of echocardiographic and biomarker parameters between children with dilated cardiomyopathy (DCM) in NYHA/Ross class I–II and class III–IV. Values are presented as mean ± SD. Abbreviations: NT-proBNP—N-terminal pro–B-type natriuretic peptide; MAPSE—mitral annular plane systolic excursion; LVEDD—left ventricular end-diastolic diameter; LVEF—left ventricular ejection fraction; LVSF—left ventricular shortening fraction; GLS—global longitudinal strain.

	DCM Group NYHA/Ross I–II	DCM Group NYHA/Ross III–IV	*p*-Value
NT-proBNP (pg/mL)	119.5 (IQR 77.5–371.3)	3500 (IQR 1706–6601)	*p* < 0.001
z-log NT-proBNP	1.40 ± 1.07	4.85 ± 1.34	*p* < 0.001
MAPSE (cm)	1.17 ± 0.38	0.88 ± 0.2	*p* < 0.05
LVEDD Z-score	2.71 ± 0.7	4.09 ± 1.53	*p* < 0.001
LV EF (%)	38.28 ± 11.48	21.68 ± 11.86	*p* < 0.001
LV SF (%)	28.22 ± 7.87	17.65 ± 8.74	*p* < 0.001
GLS (%)	−16.59 ± 5.78	−7.406 ± 2.98	*p* < 0.001

Abbreviations: NT-proBNP—N-terminal pro–B-type natriuretic peptide, MAPSE—mitral annular plane systolic excursion; LVEDD—left ventricular end-diastolic diameter; LVEF—left ventricular ejection fraction; LVSF—left ventricular shortening fraction; GLS—global longitudinal strain.

**Table 6:** Parsimonious GLS model and sensitivity analyses. OR for GLS are reported per 1% increase (less negative). Sensitivity models adjust separately for systolic BP and HR. Calibration was acceptable in all models (Hosmer–Lemeshow *p* = 0.56–0.70) with overall accuracy 88.9–89.3%. The GLS coefficient changed by ≤2% versus the primary model, indicating negligible hemodynamic confounding. * Odds ratios (OR) are expressed per 1% absolute increase in GLS.Abbreviations: GLS, global longitudinal strain; sBP, systolic blood pressure; HR, heart rate; OR, odds ratio; CI, confidence interval.

Model	GLS OR (per 1% ↑) * (95% CI)	*p*-Value	Covariate OR (95% CI)	*p*-Value
Primary (GLS only)	1.54 (1.14–2.07)	0.005	—	—
+sBP	1.55 (1.13–2.12)	0.007	1.01 (0.92–1.12)	0.798
+HR	1.53 (1.13–2.06)	0.005	1.00 (0.93–1.07)	0.973
+Age	1.51 (1.07–2.14)	0.018	0.94 (0.7–1.2)	0.63
+BMI	1.51 (1.07–2.14)	0.018	1.37 (0.82–2.31)	0.229

**Table 7:** Alternative single-predictor logistic models evaluated separately to avoid collinearity with GLS. Odds ratios are scaled for interpretability: LVEF and LVSF per 1% decrease; NT-proBNP per 100-unit increase. Higher ORs reflect greater odds of NYHA/Ross III–IV. Calibration was acceptable in all models; Arrows indicate the direction of change used for scaling. **↑** denotes that the odds ratio is expressed per unit increase in the variable, while **↓** denotes that the odds ratio is expressed per unit decrease in the variable.Abbreviations: OR, odds ratio; CI, confidence interval; LVEF, left ventricular ejection fraction; LVSF, left ventricular shortening fraction; NT-proBNP, N-terminal pro–B-type natriuretic peptide.

Predictor (Scaling)	OR (95% CI)	*p*-Value
LVEF (per 1% ↓)	1.12 (1.03–1.22)	0.009
LVSF (per 1% ↓)	1.19 (1.04–1.36)	0.011
NT-proBNP (per 100 units ↑)	≈1.11 (≈1.00–1.22)	0.013

## Data Availability

Data can be made available on request.

## Abbreviations

The following abbreviations are used in this manuscript:

DCM	Dilated cardiomyopathy
HF	Heart failure
NYHA	New York Heart Association
LV	Left ventricle
LVEF	Left ventricular ejection fraction
LVSF	Left ventricular shortening fraction
LVEDD	Left ventricular end-diastolic diameter
LVESD	Left ventricular end-systolic diameter
LVEDV	Left ventricular end-diastolic volume
SD	Standard deviation
2D-STE	Two-dimensional speckle-tracking echocardiography
EACVI	European Association of Cardiovascular Imaging
ASE	American Society of Echocardiography
NT-proBNP	N-terminal pro-B-type natriuretic peptide
MAPSE	Mitral annular plane systolic excursion
IVS	Intraventricular septum
CO	Cardiac output
SV	Stroke volume
A4C	Apical four-chamber view
A3C	Apical three-chamber view
A2C	Apical two-chamber view
GLS	Global longitudinal strain
DICOM	Digital Imaging and Communications in Medicine
SR	Strain Rate
BLS	Basal longitudinal strain
MLS	Mid-ventricular longitudinal strain
ALS	Apical longitudinal strain
IQR	Interquartile range
CI	Confidence interval
OR	Odds ratio
AUC	Area under the curve
ROC	Receiver Operating Characteristic
BMI	Body Mass index
NO	Number
YR	Years
KG	Kilograms
CM	Centimeters
BP	Blood pressure
HR	Heart rate
